# Behavioral Inhibition in Childhood as A Risk Factor for Development of Social Anxiety Disorder: A Longitudinal Study

**DOI:** 10.3390/ijerph17113941

**Published:** 2020-06-02

**Authors:** Garcia-Lopez Luis-Joaquin, Espinosa-Fernández Lourdes, Muela-Martínez José A

**Affiliations:** Department of Psychology, University of Jaen, 23071 Jaén, Spain; ljgarcia@ujaen.es (G.-L.L.-J.); jmuela@ujaen.es (M.-M.J.A.)

**Keywords:** behavioral inhibition, childhood, longitudinal, primary prevention, social anxiety disorder

## Abstract

Previous research has suggested the association between behavioral inhibition (BI) and the development of social anxiety disorder in childhood. However, there is scarce research using longitudinal methodology in Spanish-speaking populations. To cover this gap, the sample comprised 73 children ranging from six to eight years who had been examined for BI two years earlier in home and school settings. Children and their parents were administered the Anxiety Disorders Interview Schedule for DSM-5-Child and Parent Versions to assess the presence of possible anxiety disorders. The results revealed the stability of BI symptomatology over time. Data also showed that BI children were almost ten times more likely to develop social anxiety disorder two years later, compared to no-BI children. As a result, findings suggest behavioral inhibition strongly predicts social anxiety disorder, making BI a logical focus for selective preventive interventions. Therefore, screening for behavioral inhibition holds promise for primary prevention.

## 1. Introduction

Behavioral inhibition (BI) is a variable that entails shyness, withdrawal, social avoidance, anxiety and social discomfort, as well as fear in response to unfamiliar people, objects and/or situations [1,2]. BI is viewed as a prevalent temperamental trait, affecting 15%−20% of children and defined by apprehension to novelty [3,4,5,6]. Although BI is observed very early on in life [1,7], it is necessary to consider the child’s developmental period when it comes to its detection. From this perspective, BI must not be confused with the normal stranger anxiety reaction shown by seven-to eight-month-old infants, who at this stage are able to tell the difference between the attachment bond figure, usually the mother, and unfamiliar persons [8]. Spitz argued that this type of anxiety expresses good emotional/affective development and maturation, unlike the maladaptive behavior underlying BI that can be detected between four and six years of age [9] and which can continue throughout childhood, adolescence [6,10,11] and even into adulthood [12,13]. 

The assessment and detection of BI in childhood has largely been based on the information provided by parents or teachers [14], given that children are not yet able to inform about certain personal variables at such an early age [15]. However, informants’ reports commonly disagree in their perceived levels of child behavior [16].

Therefore, it is crucial to collect data from both parents and teachers to build a broad, exhaustive, complete and detailed enough profile of their behavioral repertoire [17]. The life stage at which the child starts school, namely preschool education, seems to be the ideal time to undertake their assessment. This stage of childhood is not only advanced enough for detection to still be considered early, but it is also far enough along to enable information to be gathered from different contexts, school included, and from different informants, i.e., parents and teachers [15].

Although one of the procedures most commonly used to assess BI includes paper-and-pencil tests, a review of paper-and-pencil questionnaires has revealed a lack of specific BI evaluation for preschool children. [18]. There are, however, three exceptions: a) the Parental Inhibition Scale developed by Asendorpf [10]; b) the Behavioral Inhibition Questionnaire by Bishop, Spence and McDonald [19]; and c) the Escala de Inhibición Conductual para Preescolares (Preschool Behavioral Inhibition Scale) devised by Ballespí, Jané, Riba and Domènech-Llaberia [20] and modified by Ballespí, Jané and Riba in 2012 [21]. The latter is the only one available in Spanish and covers the early childhood years.

There are many studies that correlate BI with anxiety disorders (ADs) [2,22,23,24,25,26,27,28,29,30,31,32]. These studies suggest that BI may be considered as a behavioral marker of biological vulnerability in the development of ADs [1,33,34]. A recent meta-analysis revealed that nearly half of individuals who express high levels of BI in childhood will develop an anxiety disorder sometime in their life, a four-fold increase in risk over individuals with no history of BI [35]. Despite this, there are a scarce number of longitudinal studies testing BI as a potential risk factor for the later development of social anxiety disorder (SAD). Hudson et al. [27] found that four-year-old children in their study identified with BI were significantly more likely to be diagnosed with SAD at age six in Australia. Muris et al. [36] indicated that behavioral inhibition primarily acted as a specific risk factor for the development of social anxiety symptoms during a three-year period in the Netherlands. Using retrospective data, other studies provided data suggesting BI explained 7.6% of variance for an anxiety disorder at age six, and particularly SAD (10.4% of the total variance) [37].

Symptoms of anxiety in children are highly prevalent [38,39], but researchers have stressed the need for examining risk factors in early childhood [37,40,41]. No research has been conducted so far in Spanish-speaking populations using a longitudinal approach. To cover this gap, this paper examines BI as a risk factor for the development of social anxiety disorder in particular, as well as anxiety and mood disorders for Spanish-speaking children. For the very first time, this study will include multiple informants (both teacher, father and mother) and contexts, from both school and home.

## 2. Materials and Methods 

### 2.1. Participants

The sample was composed of 73 children classified at age 4−6 years as either behaviorally inhibited (N = 22) or behaviorally uninhibited (N = 51). BI was assessed again 2 years later when participants were 6−8 years of age. The mean age for the whole sample was 6.9 years (SD = 0.6), with 33 boys (45.2%) and 40 girls (54.8%). For the BI subsample, the mean age was 6.8 years (SD = 0.5), with 10 boys (45.5%) and 12 girls (54.5%). In case of no-IB sample, the mean age was 6.9 years (SD = 0.6), with 23 boys (45.1%) and 28 girls (54.9%). No differences in gender or age between BI and no-BI children were observed.

In terms of the study’s data collection, the fathers, mothers and teachers served as informants (school and home settings). Regarding level of education, 11 children (15.1%) were in year five of pre-primary education, 40 (54.8%) in primary year one, and 22 children (30.1%) in their second year of primary education. To code the socioeconomic status of the participating subjects, the profession held by the most socioeconomically advantaged parent at the time of study. As a result, the socioeconomic status of this sample of children was middle (Hollingshead, 1975). Most informants in the family context were the children’s biological parents (95%), followed by foster parents (5%). Among them, 1% was under 25 years of age, 30.3% between 26 and 35 years, 66% between 36 and 45, and 2.7% between 46 and 55. Regarding ethnicity, 98% of the parents were Spanish citizens compared to 2% from other countries. This data is consistent with the social reality in the region according to INE data (National Statistics Institute) [42], which recorded a foreign population of 3% in the area during the study period.

### 2.2. Procedure

Participants were recruited from two public and private primary schools at the baseline, using a between-school random assignment from the educative census. Before collecting data at the baseline and 2 year follow-up, approval of the Ethical School Board and University Committees was required (RFC/IEG 2011). The families were then informed of the study’s objectives via letter and at a meeting held in the participating schools. Informed consent forms were collected at the baseline and 2-year follow-up. At the baseline, the participation rate was 89% for parents (both father and mother) and 100% for teachers. At the follow-up, all parents and children participated in the study. The investigation was carried out following the rules of the Declaration of Helsinki of 1975, revised in 2013.

### 2.3. Measures

Given the longitudinal nature of the study, children previously classified at age 4−6 years as either behaviorally inhibited or behaviorally uninhibited were assessed 2 years later when participants were 6−8 years of age to reexamine BI and assess the presence of anxiety disorders. Participants were classified as BI children based on home contexts (by father or mother report) and school settings (by teacher report). Only children surpassing the cut-off score on the Preschool Behavioural Inhibition Scale (PBIS) in both contexts were identified as BI children. 

The PBIS [20] used for assessing BI was originally developed in Spain. The measure evaluated BI when the child was faced with novel stimuli, eliciting responses from 1 to 4. It can be administered on an individual or group basis to assess children at the baseline and 2 year follow-up, lasting for approximately 5 minutes. The scale has good internal consistency, convergent and discriminant validity to be administered to parents and teachers [19,43]. Scoring within the highest 85th percentile on the PBIS was adopted as the inclusion criteria, as suggested by literature [3,4,5,6]. In this study, alpha values were 0.87 and 0.86 for parents and teachers, respectively.

The Anxiety Disorders Interview Schedule for DSM-5—Child and Parent Versions (ADIS-5-C/P) [44] assesses anxiety disorders in youth aged 6 to 17 years and is organized according to anxiety disorders included in the Diagnostic and Statistical Manual of Mental Disorders, 5th edition (DSM-5) [45]. The ADIS-5-C/P consists of comparable but separate child and parent interviews. At the follow-up, the ADIS-5-C/P was administered to children and their parents to examine the presence of ADs. A composite Index is obtained. In this study, alpha values for anxiety and mood sections ranged from 0.82 to 0.92.

### 2.4. Statistical Analysis

Analyses were performed using the statistical software package SPSS 20.0 for Windows (IBM Corp., Armonk, NY, USA). The significance level was set at *p* < 0.05. Statistically significant relationships between BI and ADs were evaluated by means of likelihood ratio (LR) testing. The effect size values for each of the significant differences found in the LR were also obtained. To achieve this, the odds ratio or probability of a condition occurring in one population group against the probability of this not happening was calculated.

## 3. Results

At the baseline, the parents’ PBIS mean score was 21.70 (range: 15−41; SD: 5.14) whereas the mean score at the follow-up was 22.90 (range: 16−33; SD: 3.44). From the teachers’ reports, the PBIS mean score was 20.74 (range: 14−42; SD: 5.79) at the baseline and 20.52 (range: 15−37; SD: 3.28) at the follow-up. No differences on age or gender were observed. The test-retest stability coefficient for PBIS, over the 2 year follow-up interval, were: r = 0.60 (parent form), and 0.40 (teacher form). Taking all of these data together, findings reveal that BI children evidenced stability on their symptomatology. 

Overall, BI did not reveal to act as a risk factor for the development of anxiety or mood disorders in children, with the exception of social anxiety disorder. Data reveal that four (18.2%) out of the 22 children classified as behaviorally inhibited developed SAD two years later, whereas only one (2%) out of the 51 no-BI children met the criteria for SAD. These results reveal a risk-sharing relationship (LR = 5.75; *p* = 0.016) between BI presence and SAD development, yielding a Relative Risk/Risk Ratio value of 9.3 (95% CI: 4.0−26.8). In other words, 4−6 year-olds identified with BI were almost ten times more likely to be diagnosed with SAD at ages 6−8 (see Table 1). Relative Risk/Risk Ratios fell into the 0.7–2.3 range for the remaining ADs, being non-significant in all cases (*p* > 0.05). Post-traumatic stress disorder and major depressive disorder were not detected in any condition.

## 4. Discussion

The study reveals a unique pattern: BI children present were 9.3 times more likely to develop SAD than their uninhibited counterparts. These findings are consistent with previous studies that link BI with SAD during childhood [28,30,31,35]. However, BI did not contribute to the development of ADs, which differ from previous data [2,23,24,25,26,27,29,32,37,46].

Our data also showed the stability of BI, in line with research suggesting BI may be viewed as a behavioral marker of biological vulnerability or role of certain parenting behaviors, paving the way for SAD [1,33,34,47]. Therefore, BI can be considered as a risk factor for SAD development, making BI a logical focus for selective preventive interventions. Screening BI could assist parents and school staff in identifying children at risk for SAD and potentially target cost-effective preventive interventions. 

As Dodd, Hudson and Rapee suggest, there are two ways BI could be used either as a marker on which to select children at risk or as a risk factor that might be changed to reduce future risk [48]. Thus, future research could focus on implementing early prevention programs for SAD in BI children. For instance, parents could benefit from: i) psychoeducation on anxiety and BI, the potential role of emotional intelligence; ii) training in those with high levels of expressed emotion (hostility, criticism and parental over-involvement); iii) parent-management techniques; and iv) being trained in cognitive and exposure techniques to implement with their offspring. Recently, the Turtle Program has been designed to treat children with elevated BI by intervening at the level of both parents and peers (indicated prevention) [49].

In addition to the possible implementation of early prevention programs addressing BI as a risk factor for SAD, future research could examine personal factors (e.g., place of origin, sex, age range) and familial factors in parents and teachers (e.g., expressed emotion, educational styles, psychopathologies) that may also be related to BI. A recent study revealed that self-reported and retrospectively assessed BI was associated with poorer social cognition in adolescence and impairment of mentalizing (MZ) in adolescence [22]. Future studies should replicate this finding by using a longitudinal methodology along with administering parents and teachers measures. Finally, future studies could collect data using behavioral measures not only from both parents and teachers but also from peer interactions as suggested by Rubin, Barstead, Smith, and Bowker [50].

This study has some limitations. First, the relatively small sample size as a result of strict criteria to collect data from all informants (father, mother and teacher) may have affected the generalizability of the results. Second, even though participants were recruited using between-school random assignment from the educative census, it was composed of heteronormative nuclear families. Future studies should try to be more inclusive. Third, future studies could explore moderating factors (e.g., emotional intelligence) that may serve as protective factors in BI children who did not develop ADs. The study also has several strengths. Despite the limited sample size, this is the first longitudinal study to explore the relationship between BI in childhood and ADs in a Spanish-speaking sample. It is also the first to consider three informants in two contexts (school and home): teachers, mothers and fathers. 

## 5. Conclusions

BI children evidence an almost 10 times higher risk of social anxiety. However, behavioral inhibition does not play a significant role in the development of anxiety disorders, such as generalized anxiety disorder, specific phobia, separation anxiety disorder, or obsessive–compulsive disorder. BI children are not significantly more likely to develop other disorders, such as dysthymia. Therefore, behavioral inhibition is a unique risk factor for the development of social anxiety disorder in childhood. 

## Figures and Tables

**Table 1:** Likelihood ratio for children with and without behavioral inhibition (BI) for each anxiety and mood disorder.

Disorder	*N* = 73			*n_B_* = 22			*n_NBI_* = 51		
	LR	*p*	N	*p*	*n_DNBI_*	*p*	*n_DNBI_*	*p*	RR
Separation anxiety disorder	1.56	0.21	8	11	4	18.2	4	7.8	2.3
Social anxiety disorder	5.75	0.02	5	6.8	4	18.2	1	2	9.3
Specific phobia	0.08	0.77	25	34.2	7	31.8	18	35.3	0.7
Generalized anxiety disorder	0.02	0.90	3	4.1	1	4.5	2	3.9	1.2
Obsessive–compulsive disorder	0.72	0.40	1	1.4	0	0	1	2	0.8
Dysthimia	0.72	0.40	1	1.4	0	0	1	2	0.8

*N* = total sample; *n_BI_* = children with BI; *n_NBI_* = children without BI; *LR* = likelihood ratio; *P* = percentage; *n* = children with disorders; *n_DBI_* = children with disorders with BI; *n_DNBI_* = children with disorders without BI; RR = Relative Risk/Risk Ratio

## References

[1] Kagan J., Reznick J., Snidman N. (1988). Biological bases of childhood shyness. Science.

[2] Vreeke L.J., Muris P., Mayer B., Huijding J., Bos A., Van Der Veen M., Raat H., Verheij F. (2012). The assessment of an inhibited, anxiety-prone temperament in a Dutch multi-ethnic population of preschool children. Eur. Child Adolesc. Psychiatry.

[3] Kagan J. (1997). Temperament and the Reactions to Unfamiliarity. Child Dev..

[4] Kagan J., Snidman N. (2004). The Long Shadow of Temperament.

[5] Kagan J., Reznick J.S., Snidman N. (1987). The Physiology and Psychology of Behavioral Inhibition in Children. Child Dev..

[6] Kagan J., Snidman N., Arcus D. (1998). Childhood Derivatives of High and Low Reactivity in Infancy. Child Dev..

[7] Kagan J., Reznick J.S., Snidman N., Gibbons J., Johnson M.O. (1988). Childhood Derivatives of Inhibition and Lack of Inhibition to the Unfamiliar. Child Dev..

[8] Spitz R. (1961). El Primer Año De La Vida Del Niño.

[9] Biederman J., Faraone S.V., Marrs A., Moore P., Garcia J., Ablon S., Mick E., Gershon J., Kearns M.E. (1997). Panic Disorder and Agoraphobia in Consecutively Referred Children and Adolescents. J. Am. Acad. Child Adolesc. Psychiatry.

[10] Asendorpf J.B. (1990). Development of inhibition during childhood: Evidence for situational specificity and a two factor model. Dev. Psychol..

[11] Pfeifer M., Goldsmith H., Davidson R.J., Rickman M. (2002). Continuity and change in inhibited and uninhibited children. Child Dev..

[12] Caspi A., Harrington H., Milne B., Amell J.W., Theodore R.F., Moffitt T.E. (2003). Children’s behavioral styles at age 3 are linked to their adult personality traits at age 26. J. Pers..

[13] Kagan J., Snidman N., Kahn V., Towsley S. (2007). The preservation of two infant temperaments into adolescence. Child Dev..

[14] Goldsmith H.H., Buss A.H., Plomin R., Rothbart M.K., Thomas A., Chess S., Hinde R.A., McCall R.B. (1987). Roundtable: What Is Temperament? Four Approaches. Child Dev..

[15] Ordóñez-Ortega A., Espinosa-Fernández L., Garcia-Lopez L., Muela-Martínez J.A. (2013). Inhibición Conductual y su Relación con los Trastornos de Ansiedad Infantil. Ter. Psicológica.

[16] Reyes A.D.L., Thomas S.A., Goodman K.L., Kundey S. (2012). Principles underlying the use of multiple informants’ reports. Annu. Rev. Clin. Psychol..

[17] Amador J.A., Forns M., Guàrdia J., Peró M. (2006). Estructura factorial y datos descriptivos del perfil de atención y del cuestionario para niños en edad escolar. Psicothema.

[18] Ballespí S., Jané M.C. (2002). ¿Cómo evaluar la inhibición conductual? Una revisión de instrumentos. Revista de Psiquiatría Infanto-Juvenil.

[19] Bishop G., Spence S.H., McDonald C. (2003). Can Parents and Teachers Provide a Reliable and Valid Report of Behavioral Inhibition?. Child Dev..

[20] Ballespí S., Jané M.C., Riba M.D., Domènech-Llaberia E. (2003). Escala de Inhibición Conductual para Preescolares, versión maestros (EICP-M): Propiedades psicométricas. Psicothema.

[21] Ballespí S., Jané M.C., Riba M.D. (2012). The Behavioural Inhibition Scale for Children Aged 3 to 6 (BIS 3–6): Validity Based on Its Relation with Observational Measures. J. Psychopathol. Behav. Assess..

[22] Ballespí S., Pérez-Domingo A., Vives J., Sharp C., Barrantes-Vidal N. (2018). Childhood behavioral inhibition is associated with impaired mentalizing in adolescence. PLoS ONE.

[23] Broeren S., Newall C., Dodd H.F., Locker R., Hudson J.L. (2014). Longitudinal investigation of the role of temperament and stressful life events in childhood anxiety. Dev. Psychopathol..

[24] Dodd H.F., Hudson J.L., Morris T., Wise C.K. (2012). Interpretation bias in preschool children at risk for anxiety: A prospective study. J. Abnorm. Psychol..

[25] Hudson J.L., Dodd H.F. (2012). Informing Early Intervention: Preschool Predictors of Anxiety Disorders in Middle Childhood. PLoS ONE.

[26] Hudson J.L., Dodd H.F., Bovopoulos N. (2011). Temperament, Family Environment and Anxiety in Preschool Children. J. Abnorm. Child Psychol..

[27] Hudson J.L., Dodd H.F., Lyneham H.J., Bovopoulos N. (2011). Temperament and Family Environment in the Development of Anxiety Disorder: Two-Year Follow-up. J. Am. Acad. Child Adolesc. Psychiatry.

[28] Lewis-Morrarty E., Degnan K.A., Chronis-Tuscano A., Pine D.S., Henderson H.A., Fox N.A. (2014). Infant attachment security and early childhood behavioral inhibition interact to predict adolescent social anxiety symptoms. Child Dev..

[29] Pahl K.M., Barrett P.M., Gullo M.J. (2012). Examining potential risk factors for anxiety in early childhood. J. Anxiety Disord..

[30] Panayiotou G., Karekla M., Panayiotou M. (2014). Direct and indirect predictors of social anxiety: The role of anxiety sensitivity, behavioral inhibition, experiential avoidance and self-consciousness. Compr. Psychiatry.

[31] Papachristou H., Theodorou M., Neophytou K., Panayiotou G. (2018). Community sample evidence on the relations among behavioural inhibition system, anxiety sensitivity, experiential avoidance, and social anxiety in adolescents. J. Context. Behav. Sci..

[32] Sportel B.E. (2013). Adolescents at Risk for Social and Test Anxiety: Who are at Risk and How Can We Help?. Ph.D. Thesis.

[33] Biederman J., Hirshfeld-Becker D.R., Rosenbaum J.F., Hérot C., Friedman D., Snidman N., Kagan J., Faraone S.V. (2001). Further Evidence of Association Between Behavioral Inhibition and Social Anxiety in Children. Am. J. Psychiatry.

[34] Hirshfeld D.R., Rosenbaum J.F., Biederman J., Bolduc E.A., Faraone S.V., Snidman N., Reznick J.S., Kagan J. (1992). Stable Behavioral Inhibition and Its Association with Anxiety Disorder. J. Am. Acad. Child Adolesc. Psychiatry.

[35] Clauss J., Blackford J.U. (2012). Behavioral inhibition and risk for developing social anxiety disorder: A meta-analytic study. J. Am. Acad. Child Adolesc. Psychiatry.

[36] Muris P., Van Brakel A.M.L., Arntz A., Schouten E. (2010). Behavioral Inhibition as a Risk Factor for the Development of Childhood Anxiety Disorders: A Longitudinal Study. J. Child Fam. Stud..

[37] Paulus F.W., Backes A., Sander C.S., Weber M., Von Gontard A. (2014). Anxiety Disorders and Behavioral Inhibition in Preschool Children: A Population-Based Study. Child Psychiatry Hum. Dev..

[38] Essau C.A., Gabbidon J., Essau C.A., Ollendick T.H. (2013). Epidemiology, Comorbility and Mental Health Service Utilization. The Wiley-Blackwell Handbook of the Treatment of Childhood and Adolescent Anxiety.

[39] Lavigne J., LeBailly S.A., Hopkins J., Gouze K.R., Binns H.J. (2009). The Prevalence of ADHD, ODD, Depression, and Anxiety in a Community Sample of 4-Year-Olds. J. Clin. Child Adolesc. Psychol..

[40] Egger H., Angold A. (2006). Common emotional and behavioral disorders in preschool children: Presentation, nosology, and epidemiology. J. Child Psychol. Psychiatry.

[41] Behavioral Inhibition (2018). Encyclopedia of the Sciences of Learning.

[42] Instituto Nacional de Estadística (España) Padrón Población por Municipios. http://https://www.ine.es/index.htm.

[43] Espinosa-Fernandez L., Garcia-Lopez L.J., Muela J.A., Ordoñez-Ortega A. Understanding children with Behavioral Inhibition: Multinformant approach in educational and family contexts. Psychologica.

[44] Albano A.M., Silverman W.K. (2020). The Anxiety Disorders Interview Schedule for Children for DSM-5, Child and Parent Versions.

[45] Battle D.E. (2013). Diagnostic and Statistical Manual of Mental Disorders (DSM). CoDAS.

[46] White L.K., Degnan K.A., Henderson H.A., Pérez-Edgar K., Walker O.L., Shechner T., Leibenluft E., Bar-Haim Y., Pine D.S., Fox N.A. (2017). Developmental Relations Among Behavioral Inhibition, Anxiety, and Attention Biases to Threat and Positive Information. Child Dev..

[47] Rubin K., Burgess K.B., Hastings P.D. (2002). Stability and social-behavioral consequences of toddlers’ inhibited temperament and parenting behaviors. Child Dev..

[48] Dodd H.F., Hudson J.L., Rapee R.M. (2017). Temperament in Youth Internalizing Disorders. Treatments for Psychological Problems and Syndromes.

[49] Barstead M.G., Danko C.M., Chronis-Tuscano A., O’Brien K.A., Coplan R.J., Rubin K. (2018). Generalization of an Early Intervention for Inhibited Preschoolers to the Classroom Setting. J. Child Fam. Stud..

[50] Rubin K., Barstead M.G., Smith K.A., Bowker J.C. (2018). Peer Relations and the Behaviorally Inhibited Child. Behavioral Inhibition.

